# On the relevance of double tax treaties

**DOI:** 10.1007/s10797-019-09570-9

**Published:** 2019-09-21

**Authors:** Kunka Petkova, Andrzej Stasio, Martin Zagler

**Affiliations:** 1 WU Vienna University of Economics and Business, Welthandelsplatz 1, Building D3, 1020 Vienna, Austria; 2 UPO University of Eastern Piedmont, Via Perrone 18, 28100 Novara, Italy; 3 WU Vienna University of Economics and Business, Welthandelsplatz 1, Building D4, 1020 Vienna, Austria

**Keywords:** Double tax treaties, FDI, Treaty shopping, Tax treaty network, F21, F23, F53, H25, H26, H73, H87, K34

## Abstract

This paper investigates the effects of double tax treaties (DTTs) on foreign direct investment (FDI) after controlling for their relevance in the presence of treaty shopping. DTTs cannot be considered a bilateral issue, but must be viewed as a network. We define tax distance as the cost of channelling corporate income from one country to another and, by considering treaty shopping through intermediate jurisdictions, we calculate the shortest (i.e. the cheapest) distance between any two countries. We show that relevant tax treaties—which reduce the direct tax distance both over domestic law and the entire existing treaty network—will increase FDI by about 18%.

## Introduction

Traditionally, double tax treaties (DTTs) served as an important policy tool to promote international economic activity by preventing international double taxation. However, despite the growing number of contributions, the empirical evidence on the effects of double tax treaties on bilateral FDI remains inconclusive (Blonigen and Davies 2004; Egger et al. 2006; Egger and Merlo 2011; Blonigen et al. 2014).

The well-intended motivation to eliminate double taxation has created a highly complex network of DTTs that span the globe, with often unforeseen consequences (Easson 2000). While preventing international double taxation, DTTs shift taxing rights from capital-importing countries to capital-exporting countries, denying investors the benefits from lower source taxation (Braun and Zagler 2014). Moreover, in order to avoid high host-country withholding taxes on outgoing passive income, many multinational companies divert FDI via a third country with a more favourable tax treaty, a practice that has been labelled treaty shopping in the literature (Dyreng et al. 2015). The OECD highlights that treaty shopping is one of the most significant sources of concerns regarding the Base Erosion and Profit Shifting (BEPS) project (OECD 2015).

Against this background, this paper investigates the effects of double taxation treaties on foreign direct investment (FDI) controlling for the possibility of treaty shopping that might give multinational companies benefits, such as lower or no withholding taxes. However, in contrast to the previous literature, instead of treating tax treaties as a binary variable, thereby ignoring their complexity and their domestic and international interactions, our study analyses the effects of DTTs in a richer setting that goes beyond their binary treatment. By doing so, we are able to evaluate their relevance given the entire tax treaties network and allow for a differential effect on FDI. Furthermore, as opposed to prior studies that concentrate on a single country or a single year and ignore any changes in the tax treaties network over time, we consider the full period between 2005 and 2012, which allows for sufficient variation under domestic law and in the tax treaties network of 138 countries.

In line with Barrios et al. (2012), we interpret the international tax system as a network where the tax distance between two countries is defined as the cost of channelling corporate income from one country to another in terms of taxes to be paid. In particular, the tax cost between two countries consists of corporate income taxes to be paid in the host country, a non-resident withholding tax on the income of the subsidiary and corporate income taxes in the home country. We account for treaty shopping and calculate the shortest (i.e. the cheapest) distance between any two countries allowing for corporate income to be channelled through one or more intermediate jurisdictions. Our main hypothesis is that only *relevant* tax treaties—i.e. tax treaties that offer investors a financial advantage over the conditions under domestic law and given the entire existing tax treaties network—lead to more immediate home to host-country FDI.

We indeed find that DTTs that offer investors a financial advantage over the conditions under domestic law increase FDI, whereas DTTs that do not provide this benefit have no impact on FDI. This effect is bigger for DTTs that offer a financial advantage over all other tax treaties in the network. Furthermore, for these network effects we differentiate between tax-minimising indirect routes with one and two intermediate jurisdictions. We show that when the tax-minimising indirect route involves only one conduit, tax treaties that do not improve conditions for investors have no effect on the bilateral FDI between home and host countries. This case is indistinguishable from not having a treaty. If the indirect route involves two conduits and is therefore more cumbersome, a DTT can lead to more direct FDI even if it is not offering a financial advantage. Finally, we find that the effects on FDI increase with reductions in the direct tax distance below the minimum one in the network.

Our paper contributes to the existing literature in two important ways: identifying the impact of DTTs on FDI and explicitly taking into account treaty shopping. First and foremost, we address the so far empirically mixed results in the prior literature by showing that the reason for this inconclusiveness comes from the binary treatment of the DTTs and not accounting for the tax environment in which they occur.

The economic effects of DTTs have been analysed in numerous studies. Using 1982–1992 OECD data on the aggregate stocks and flows of bilateral FDI, Blonigen and Davies (2005) find that new tax treaties have a strong negative impact on FDI. Blonigen and Davies (2004) confirm these results using US data. The authors attribute their results to DTTs reducing tax evasion, at least in the short run. For a sample of 67 DTTs and aggregate bilateral outward FDI between OECD countries from 1985 to 2000, Egger et al. (2006) find a negative average treatment effect of DTTs on FDI using different matching estimators and focusing on difference in differences. Baker (2014) uses a similar estimation strategy and shows that tax treaties do not have any effect on FDI.

Against all these results, Neumayer (2007) finds robust empirical evidence that DTTs increase FDI to developing countries. However, when the author splits developing countries into low-income and middle-income countries, he finds that DTTs are effective only in the group of middle-income countries. Using micro-data on multinational-firm activity, Egger and Merlo (2011) argue that DTTs have a positive effect on foreign investments of multinational firms. Yet, their findings are limited to German multinational enterprises (MNEs). Similarly, using the United States Bureau of Economic Analysis firm-level data, Blonigen et al. (2014) find a positive effect of DTTs on foreign direct investment, but only when accounting for the firms’ use of differentiated inputs. These (multinational) firms benefit from treaty provisions establishing guidelines for resolving disputes between taxation authorities. In contrast, firms that use more homogenous inputs are on average less likely to see any significant effect.

A closely related stream of the literature considers the effects of DTTs on the location decision of multinational firms. Using micro-data from Sweden between 1965 and 1998, Davies et al. (2009) find a positive effect of DTTs on a multinational firm’s decision to locate the first affiliate in a treaty country. The authors argue that the positive effect of DTTs comes from the reduced investment uncertainty. Marques and Pinho (2014) analyse the extent to which tax treaties influence the number of new foreign subsidiaries incorporated by European multinationals between 2000 and 2009. Using two measures of tax treaties—a binary variable and a measure of cross-border effective tax rate—they provide evidence that tax treaties induced a positive and significant impact on the number of foreign subsidiaries incorporated in the last decade. However, neither of these contributions provides conclusive evidence on the effect of DTTs on FDI.

Prior literature offers several explanations for these ambiguous and inconclusive results. As argued by Owens (1962), and later pointed out by Davies (2004) and Baker (2014), for given tax rates, double taxation can be relieved just as easily unilaterally as through a bilateral tax treaty. In particular, since most capital-exporting countries already offer tax credits or exemptions, treaties have only a very limited role in avoiding double taxation. More generally, Bösenberg et al. (2016) suggest that the impact of DTTs depends on their content (e.g. which method of double tax relief is specified in a treaty or whether a treaty includes provisions on exchange of information) and the economic environment in which they occur (e.g. the profitability of bilateral multinational activity in the absence of a treaty; domestic corporate and withholding tax rates; and the unilateral method of double taxation relief). Meanwhile, the vast majority of the existing literature treats DTTs as a binary variable, thereby ignoring their complexity and their domestic and international interactions.

Our study addresses this gap in the literature and analyses the effects of double tax treaties in a richer setting that goes beyond their binary treatment. In particular, our paper reconciles the macro-level FDI data with the institutional framework common for studies using firm-level data.

In essence, micro-level studies are either better able to control for the tax environment in which DTTs occur (e.g. Marques and Pinho 2014, ), or the treaty impact in the presence of firm heterogeneity (Davies et al. 2009; Blonigen et al. 2014). Among these, our work resembles the Marques and Pinho (2014) study, which takes into account the effect of DTTs on the combined effective tax rates. However, we advance their work by considering the benefit of every DTT on combined effective tax rate relative to the conditions under domestic law and all other tax treaties in the network by allowing for the possibility of treaty shopping. Finally, we demonstrate that our results are not driven by sample size, time series, estimation methodology and omitted variable bias and, as such, are directly comparable to the results of the previous literature.

Second, we build on the work of Mintz and Weichenrieder (2010), Dreßler (2012) and Weyzig (2013) and analyse the effects of DTTs on FDI in the presence of treaty shopping. Mintz and Weichenrieder (2010) construct the chains of corporate structure for German multinationals across various countries for the year 2001 and relate these structures to the underlying fiscal motives. The level of withholding taxes is found to be important in determining which countries are used as a platform for investments. More specifically, higher bilateral withholding taxes to and from Germany substantially increase the probability that outward and inward FDI is diverted via a third country.

Dreßler (2012) and Weyzig (2013) reach similar conclusions: tracing the group structures of multinationals across 58 countries in the years 1996–2008 and analysing to what extent these structures are tax-efficient Dreßler shows that the level of withholding taxes between two group members is found to be important in determining the probability of an indirect participation. Weyzig (2013) uses micro-data from Dutch special purpose entities to analyse the geographical patterns and the structural determinants of FDI diversion. He finds that tax treaties are a key determinant of FDI routed through the Netherlands with the reduction of dividend withholding tax rates as the driving mechanism.

Taking a network approach to the study of DTTs, we also present an alternative and more accurate methodology than usual for the literature that adopts a network approach to investigate the tax treaties network (van’t Riet and Lejour 2018; Hong 2018). In order to avoid high host-country withholding taxes on outgoing passive income, many multinational companies divert FDI via a third country with a more favourable tax treaty. According to Stephen Shay, if a country has several tax treaties, MNEs will take advantage of the *“worst”* one: i.e. *“the treaty that allows for the most tax avoidance and establishes tax-treaty linkages to jurisdictions with very low or even zero corporate income tax rates”* (Brumby and Keen 2016). It is plausible that DTTs have a different effect on investment depending on whether investors consider the direct route as a viable investment channel. Therefore, we estimate the minimum tax cost between any two countries in our sample and evaluate the benefit of every single DTT relative to the minimum tax distance between the two countries with the respective tax treaty. This allows us to differentiate between different types of DTTs according to their benefit and position in the network instead of a simple binary treatment. Only then, we empirically estimate the impact of tax treaties on FDI.

We conduct this analysis using a panel of 138 countries and their corresponding tax treaties network between 2005 and 2012, allowing for sufficient variation under domestic law and in the tax treaties network as opposed to prior studies that concentrate on a single country or a single year. Finally, in contrast to the previous literature that assumes nominal, average or minimum corporate income tax rates to be credited in conduit situations, we do not use such approximations. Instead, we consider the actual taxes paid en route. By doing so, we are able to measure the impact of DTTs on combined effective tax rates given the entire tax treaties network and estimate the corresponding effect on FDI.

The remainder of this paper is structured as follows: Sect. 1 provides the theoretical background. In Sect. 2 we discuss our sample and research methodology. We present our main results in Sect. 3 with a variety of robustness tests in Sect. 4. We discuss the forces underpinning our results in Sect. 5. Section 6 concludes.

## Theoretical background

In line with Barrios et al. (2012), we capture the features of the international tax system by measuring the tax distance between two countries, where tax distance is defined as the cost of channelling corporate income from one to another in terms of taxes to be paid. In particular, the tax cost of a multinational enterprise (MNE) consists of corporate income taxes to be paid in the country of residence of the parent as well as corporate income taxes and non-resident withholding taxes on the income of the subsidiary. Depending on the relief method (*rm*) applied in the resident (home) country *R* on income from source (host) country *S*, the combined effective tax rate $t_{\mathrm{SR}}(rm)$ for the multinational company can be defined as:

1 $$\begin{aligned} t_{\mathrm{SR}}\,(\mathrm{no\,relief})= & {} 1-(1-t_{\mathrm{S}})(1-w_{\mathrm{SR}})+t_{\mathrm{R}}-t_{\mathrm{S}}t_{\mathrm{R}} \end{aligned}$$

2 $$\begin{aligned} t_{\mathrm{SR}}\,(\mathrm{deduction})= & {} 1-(1-t_{\mathrm{S}})(1-w_{\mathrm{SR}})(1-t{_R}) \end{aligned}$$

3 $$\begin{aligned} t_{\mathrm{SR}}\,(\mathrm{direct\,credit})= & {} \max \{1-(1-t_{\mathrm{S}})(1-w_{\mathrm{SR}}),1-(1-t_{\mathrm{S}})(1-t_{\mathrm{R}})\} \end{aligned}$$

4 $$\begin{aligned} t_{\mathrm{SR}}\,(\mathrm{indirect\,credit})= & {} \max \{1-(1-t_{\mathrm{S}})(1-w_{\mathrm{SR}}),t_{\mathrm{R}}\} \end{aligned}$$

5 $$\begin{aligned} t_{\mathrm{SR}}\,(\mathrm{exemption})= & {} 1-(1-t_{\mathrm{S}})(1-w_{\mathrm{SR}}) \end{aligned}$$

where $t_{\mathrm{S}}$ is the corporate tax rate in the source country, $t_{\mathrm{R}}$ the corporate tax rate in the residence country and $w_{\mathrm{SR}}$ the non-resident withholding tax on the income of the subsidiary.

About half of the countries in our sample operate an exemption system under which foreign dividends are not taxed in the residence country (Eq. 5)—see also Table 3.^1^ Other countries subject the received dividends to residence country taxation at the corporate tax rate $t_R$. Most of these countries avoid double taxation by crediting the taxes paid in the source jurisdiction on the amount of distributed dividends (Eq. 3). Such credit is usually limited to corporate taxes due in the residence country. In some cases, also an indirect credit for the underlying corporate taxes is offered (Eq. 4). Alternatively, a small number of countries do not exempt, nor credit foreign taxes, but instead allow them to be deducted as a business expense (Eq. 2). Finally, some (especially less developed) countries do not provide for any form of double tax relief (Eq. 1). The received dividends are then subject to full double taxation.

We also consider the possibility of an indirect repatriation of dividends, i.e. through a third (conduit) country *C*. It is rational for the MNE to choose the indirect route over the direct route, *ceteris paribus*, when its costs in terms of taxes are lower (Mintz and Weichenrieder 2010; Weyzig 2013; van’t Riet and Lejour 2018). Since the corporate income tax of the source country $t_S$ is always paid, irrespective of the relief method, we can define the direct tax distance $d_{\mathrm{SR}}$ between source country *S* and residence country *R* based only on the relevant withholding tax rate and the corporate income tax of the residence country. Depending on the relief method, the combined effective tax rate can be then defined as $t_{\mathrm{SR}}=1-(1-t_S)(1-d_{\mathrm{SR}})$, where $d_{\mathrm{SR}}$ accounts for the tax “distance” between the two countries measured in taxes paid en route:

6 $$\begin{aligned} d_{\mathrm{SR}}\,(\mathrm{no\,relief})= & {} t_{\mathrm{R}}+w_{\mathrm{SR}} \end{aligned}$$

7 $$\begin{aligned} d_{\mathrm{SR}}\,(\mathrm{deduction})= & {} t{_R}(1-w_{\mathrm{SR}})+w_{\mathrm{SR}} \end{aligned}$$

8 $$\begin{aligned} d_{\mathrm{SR}}\,(\mathrm{direct\,credit})= & {} \max \{w_{\mathrm{SR}},t_{\mathrm{R}}\} \end{aligned}$$

9 $$\begin{aligned} d_{\mathrm{SR}}\,(\mathrm{indirect\,credit})= & {} \max \{w_{\mathrm{SR}},(t_{\mathrm{R}}-t_{\mathrm{S}})/(1-t_{\mathrm{S}})\} \end{aligned}$$

10 $$\begin{aligned} d_{\mathrm{SR}}\,(\mathrm{exemption})= & {} w_{\mathrm{SR}} \end{aligned}$$

**Table 1:** International host- and home-country taxation

Method of double tax relief:	No relief	Deduction	Direct credit	Indirect credit	Exemption
*Host-country taxation*					
Profit of the subsidiary	100	100	100	100	100
Source CIT: 20% (*a*)	20	20	20	20	20
Dividend distribution	80	80	80	80	80
Withholding tax: 10% (*b*)	8	8	8	8	8
*Home-country taxation*					
Dividend received	72	72	72	72	72
Taxable base	80	72	80	100	72
Residence CIT: 50%	40	36	40	50	0
Foreign tax credit	0	0	8	28	0
Net corporate income tax (*c*)	40	36	32	22	0
Host-country CIT (*a*)	20	20	20	20	20
Taxes on repatriation (*b* + *c*)	48	44	40	30	8
Tax distance: $d_{\mathrm{SR}}$	60%	55%	50%	37.5%	10%
Combined effective tax rate: $t_{\mathrm{SR}}$	68%	64%	60%	50%	28%
After-tax profit	32	36	40	50	72

Table 1 illustrates the calculation of host-country corporate income tax (CIT), tax distance, combined effective tax rate and after-tax profits under each of the five relief methods considered in the case of gross profits of 100, host CIT of 20%, home CIT of 50% and a withholding tax rate of 10%.

It follows that the condition for treaty shopping is that total taxes over the indirect route are less than over the direct one, i.e. $1-(1-d_{\mathrm{SC}})(1-d_{\mathrm{CR}})<d_{\mathrm{SR}}$ where the total tax distance with an initial host $k=1$ and final destination $k=n$ equals $1-\sum _{k=2}^{n}(1-d_{k-1,k})$ (van’t Riet and Lejour 2018). Accounting for the possibility of an indirect repatriation of dividends, the effective tax rate on overseas profits is the minimum between the effective tax rate on a direct route and on the indirect route.

Finally, in a one-period model, where all profits are repatriated, it can be shown theoretically that FDI is decreasing in the relative effective tax rate *T* where $1-T=(1-t_{\mathrm{SR}})/(1-t_{\mathrm{R}}) = (1-d_{\mathrm{SR}})(1-t_{\mathrm{S}})/(1-t_{\mathrm{R}})$, where $t_{\mathrm{SR}}$ is again the effective tax rate on overseas profits and $t_{\mathrm{R}}$ the effective tax rate on domestic profits (Davies 2003, 2004). As both source and residence tax rates will be picked up by home- and host-country fixed effects in an empirical estimation, in the subsequent analysis we focus on tax distances.

## Data and network analysis

### Data

In order to construct our network analysis, we collect tax data for a sample of 138 countries between the years 2005 and 2012.^2^ Our main source of data on the domestic and international tax system is the IBFD Global Corporate Tax Handbooks for the years 2009–2012 and the IBFD Online Tax Platform. For the countries included in the Global Corporate Tax Handbooks, we collect information on the domestic tax system and, in particular, on taxation of foreign income (including the methods of double tax relief), as well as domestic corporate and withholding tax rates from the respective yearbook.^3^ To the extent that a country is not available in a Global Corporate Tax Handbook, we consult the closest to the missing year data source for the taxation of foreign income, including the IBFD Online Tax Platform, and, unless indicated otherwise, assume the same method of taxation of foreign income for the missing years.

Moreover, we update all domestic corporate and withholding tax rates with the EY (Ernst and Young) Corporate Tax Guides if the IBFD data are not available for a particular year. For instance, for the years 2005–2008, the EY Corporate Tax Guides are our only source of data on domestic corporate and withholding tax rates. We further hand-collect the relevant withholding tax rates and methods of double tax relief from the respective DTTs and applicable protocols. In addition, as the treaty network is subject to four types of changes, we check when new treaties become effective; if treaties have been terminated at a later point in time; if the conditions of the treaties have been changed through protocols in the following years; and if the conditions of the treaties have been altered through amendments in domestic law.^4^ Overall, we consult more than 3000 tax treaties that became effective before 2013 and around 300 accompanying protocols.

We obtain data on bilateral inward FDI stocks between 2005 and 2012 from the UNCTAD (United Nations Conference on Trade and Development) database, and we invert them to measure the investment from the home to the host country. In the presence of FDI diversion via a third country, we would ideally want to observe the indirect investment from the home to the host country via the conduit country. However, the available data report only the immediate home- to host-country FDI stocks. Therefore, we can only estimate the impact of DTTs on these immediate home- to host-country FDI stocks.^5^ Finally, the information on bilateral investment treaties (BITs) is from the Investment Policy Hub of UNCTAD.

### Network analysis

Recent contributions by van’t Riet and Lejour (2018) and Hong (2018) employ a network approach to study the centrality of countries in the tax treaties network and, respectively, the structure of tax-minimising (direct and indirect) investment routes. Both studies analyse the tax treaties network for a single year and ignore any changes in the tax treaties network over time. While Hong (2018) uses a simple computational algorithm, van’t Riet and Lejour (2018) use an adapted version of the Floyd–Warshall shortest path algorithm to estimate these tax-minimising investment routes. However, both approaches either underestimate or overestimate the potential for tax treaty shopping.^6^

We take a different approach and develop a Visual Basic Application (VBA) tool to recalculate the tax distance for every possible combination of host, home and intermediate countries.^7^ In this way, we can take into account the actual taxes paid in the jurisdiction before the one receiving the dividends—typically the intermediate jurisdiction—instead of nominal or world-average corporate tax rates. The single limitation of our approach is that we restrict the number of possible intermediate jurisdictions to two in order to avoid long computation time of the analysis. However, this may not be an unrealistic assumption, as Mintz and Weichenrieder (2010) show that only 0.2% of German multinational firms use cross-border group structures with three or more pass-through entities. Moreover, when we analyse our network using the Floyd–Warshall algorithm (allowing for an unlimited number of conduits), we do not find any indirect connection with three or more intermediate jurisdictions that would further reduce the tax distance between any two countries in our sample. For this reason too, we believe that our approach is superior to the Floyd–Warshall algorithm and allows for a more accurate network analysis.

For every year, we update the tax treaties network with all relevant changes. In particular, we account for changes in the provisions of tax treaties through amending protocols and for changes in the provisions of the tax treaties through changes under domestic law (for tax treaties that refer to conditions under domestic law); we add new tax treaties that become effective and remove tax treaties that have been terminated or replaced by new ones in the course of the year being analysed. We assume a fully owned subsidiary engaged in an active course of business and consider only domestic anti-abuse provisions.^8^ Specifically, we account for higher withholding taxes upon dividend distributions to tax havens and clauses that subject the subsidiary or the parent company to a minimum rate of CIT.

Several countries in our sample levy a higher withholding tax on dividends when these are distributed to a parent located in a tax haven. Because most of the available domestic tax havens lists are not comprehensive, we adopt a common tax haven list for all countries in our sample across the entire time period (Dyreng and Lindsey 2009).^9^ In accordance with the majority of these domestic provisions, we exclude the anti-abuse treatment when a DTT is in place. Similarly, several countries in our sample adapt a subject-to-a-minimum-tax clause as a condition for claiming the benefits of participation exemption and exemption from withholding tax on dividends.^10^ Since we observe what corporate tax rates the subsidiary and the parent company are subject to, we can easily control for this condition.

**Fig. 1:**
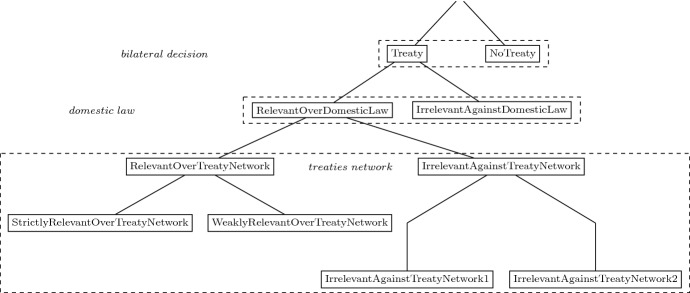
Identification strategy

We describe the entire international network of double tax treaties with a set of tax treaty dummies depicted in Fig. 1 and a measure of bilateral taxes summarised in Table 2. Our first variable is a dummy that verifies whether a DTT between two countries is present, *Treaty*. This is the standard variable used in the previous literature. Country pairs that did not conclude a treaty will be our reference category throughout our estimations. For every year in our sample, we then measure the direct tax distance between any two countries taking into account a possible tax treaty between these two countries, *DirectTaxDistance*.

Measuring the direct tax distance permits us to distinguish between treaties. First, we define *RelevantODL* tax treaties as tax treaties that reduce the effective tax rate on overseas profits below the one under domestic law of the source and residence countries. For example, in the year 2012, ignoring the bilateral tax treaty, the tax distance between Argentina as the home state and Belgium as the host state is 25% under the domestic law of both countries. However, the applicable DTT reduces the direct tax distance to approximately 1.5%. Hence, the tax treaty is *relevant* relative to domestic law provisions. We label tax treaties that do not provide for this benefit as *IrrelevantADL*. Further, we expect *RelevantODL* DTTs to increase the bilateral FDI and *IrrelevantADL* to have no effect.

The second innovative element in our analysis is to identify whether an indirect route exists along which the tax distance would be reduced as opposed to the direct route.^11^ For example, in 2012, a South African parent company investing directly in a US subsidiary had to pay 5% tax on distribution of dividends after considering the tax treaty between both countries. However, if the same investment is made through a conduit company in the Netherlands, the tax cost can be reduced to 0%.

**Table 2:** Tax treaty variables

Variable	Condition	Description
*IrrelevantAgainstDomesticLaw(IrrelevantADL)*	$d_{\mathrm{SR}} \ (domestic \ law) \le d_{\mathrm{SR}} \ (tax \ treaty)$	Direct tax distance under domestic law less or equal to direct tax distance under tax treaty
*RelevantOverDomesticLaw(RelevantODL)*	$d_{\mathrm{SR}} \ (tax \ treaty) < d_{\mathrm{SR}} \ (domestic \ law)$	Direct tax distance under tax treaty lower than direct tax distance under domestic law
*RelevantOverTreatyNetwork* (*RelevantOTN)*	$d_{\mathrm{SR}} \ (tax \ treaty) \le 1-(1 - d_{\mathrm{SC}})(1 - d_{\mathrm{CR}})$	Direct tax distance under tax treaty less or equal to minimum indirect tax distance
*StrictlyRelevantOverTreatyNetwork* (*StrictlyRelevantOTN)*	$d_{\mathrm{SR}} \ (tax \ treaty) < 1 - (1 - d_{\mathrm{SC}})(1 - d_{\mathrm{CR}})$	Direct tax distance under tax treaty less than minimum indirect tax distance
*WeaklyRelevantOverTreatyNetwork* (*WeaklyRelevantOTN)*	$d_{\mathrm{SR}} \ (tax \ treaty) = 1 - (1 - d_{\mathrm{SC}})(1 - d_{\mathrm{CR}})$	Direct tax distance under tax treaty equal to minimum indirect tax distance
*IrrelevantAgainstTreatyNetwork* (*IrrelevantATN)*	$1 - (1 - d_{\mathrm{SC}})(1 - d_{\mathrm{CR}}) < d_{\mathrm{SR}} \ (tax \ treaty)$	Minimum indirect tax distance lower than direct tax distance under tax treaty
*IrrelevantAgainstTreatyNetwork1* (*IrrelevantATN1)*	$1 - (1 - d_{\mathrm{SC}_{1}})(1 - d_{\mathrm{C}_{1}\mathrm{R}}) < d_{\mathrm{SR}} \ (tax \ treaty)$	Minimum indirect tax distance with one conduit lower than direct tax distance under tax treaty
*IrrelevantAgainstTreatyNetwork2* (*IrrelevantATN2)*	$1 - (1 - d_{\mathrm{SC}_{1}})(1 - d_{\mathrm{C}_{1}\mathrm{C}_{2}})(1 - d_{\mathrm{C}_{2}\mathrm{R}}) < d_{\mathrm{SR}} \ (tax \ treaty) \le 1 - (1 - d_{\mathrm{SC}_{1}})(1 - d_{\mathrm{C}_{1}\mathrm{R}})$	Direct tax distance under tax treaty less or equal to minimum indirect tax distance with one conduit, but higher than minimum indirect tax distance with two conduits

*Abbreviated variable names in brackets next to the corresponding variables*

Once we estimate the minimum direct and indirect tax cost between any two countries, we ask whether *RelevantODL* tax treaties remain relevant also considering the entire treaty network. We define *RelevantOTN* tax treaties as tax treaties that reduce the effective tax rate on overseas profits not only below the one under domestic law, but also to or below the minimum one in the network. In the Argentina–Belgium example above, the lowest possible tax distance when channelling income through the network is 12.5%.^12^ In the absence of the bilateral tax treaty, the MNE has a tax incentive to choose the indirect route over the direct one. However, with a direct tax distance of only 1.5%, the DTT between Argentina and Belgium takes away the advantage of the indirect one and further reduces the minimum tax distance between the two countries by 11 percentage points.

By contrast, the *IrrelevantATN* dummy indicates tax treaties that reduce the direct tax distance, but not the minimum (indirect) tax distance between the source and residence countries. Consider the case of Argentina as the home country and Germany as the host state. In 2012, the direct tax distance between the two countries is about 26.4% under their domestic law, while the minimum tax distance through the network is 12.5%. Thus, also in this case, we expect the MNE to tax-prefer the indirect route rather than the direct one. Moreover, the DTT between the two countries reduces the direct tax distance to 21.25%, which is still higher than the minimum tax distance through the network. As a result, the tax treaty between Argentina and Germany is irrelevant to the MNE’s decision to invest via a third country. To the extent that MNEs make use of treaty shopping opportunities, we expect *RelevantOTN* DTTs to have a bigger effect on FDI than *IrrelevantATN* tax treaties.

We can further decompose the *RelevantOTN* dummy and differentiate between relevant DTTs that are strictly better than the tax treaties network—*StrictlyRelevantOTN*—and relevant DTTs that just cut the tax cost of the direct route to the minimum in the network, *WeaklyRelevantOTN*. In theory, *StrictlyRelevantOTN* tax treaties should stimulate FDI between two countries for two reasons. First, firms may relocate investments from the indirect route to the direct route or invest directly where they did not invest via a conduit company despite its tax benefit in the absence of the DTT. Second, firms would also benefit from a lower overall tax burden, and this should increase FDI. Presuming non-negligible costs to treaty shopping, *WeaklyRelevantOTN* tax treaties may increase FDI between the home and host states if firms relocate investments from the indirect route to the direct route.

We can also narrow down the *IrrelevantATN* dummy and distinguish between irrelevant tax treaties where the tax-minimising indirect investment route involves one conduit country—*IrrelevantATN1*—or two conduit countries—*IrrelevantATN2*. If treaty shopping is costly, MNEs may be less likely to use more complex investment structures. Accordingly, we expect the effects of irrelevant DTTs on direct FDI to increase with the number of intermediate jurisdictions needed to set up the tax-minimising indirect route.

### Summary statistics

Our sample consists of 138 countries between 2005 and 2012, which corresponds to 18,906 unique country pairs in each sample year.^13^ However, due to missing economic data, the econometric analysis covers only 133 countries. Table 3 summarises the characteristics of the international tax network, while Table 4 gives an overview of the estimation sample summary statistics.

**Table 3:** International tax network

		2005	2012
Bilateral taxation (in the absence of DTTs):	No relief	10.4%	8.9%
	Deduction	5.7%	5.8%
	Direct credit	28.6%	27.1%
	Indirect credit	10.7%	8.8%
	Exemption	44.6%	49.4%
Bilateral taxation (in the presence of DTTs):	No relief	9.4%	7.6%
	Deduction	5.2%	5.1%
	Direct credit	28.8%	27.6%
	Indirect credit	11.2%	9.4%
	Exemption	45.4%	50.3%
Shortest distance:	Direct	53.9%	52.1%
	One conduit	35.5%	37.2%
	Two conduits	10.6%	10.7%
Number of zero tax distance connections:		7213	8907
Share of zero tax connections:	Direct	48.5%	46.1%
	One conduit	41.8%	43.3%
	Two conduits	9.7%	10.6%
Number of country pairs with an effective tax treaty:		3487	4264
Number of effective tax treaties per type:	*IrrelevantADL*	1987	2361
	*RelevantODL*	1500	1903
	*RelevantOTN*	733	890
	*StrictlyRelevantOTN*	323	311
	*WeaklyRelevantOTN*	410	579
	*IrrelevantATN*	767	1013
	*IrrelevantATN1*	723	921
	*IrrelevantATN2*	44	92

Percentages rounded to one decimal point

**Table 4:** Summary statistics estimation sample

Variable	*n*	Mean	SD	Min	Max
*FDI stocks* (in millions US dollars)	31,370	3905	21,496	0	592,273
*BIT*	31,370	0.4041	0.4907	0	1
*DirectTaxDistance*	31,370	0.1172	0.1184	0	.66
*Treaty*	31,370	0.5468	0.4978	0	1
*IrrelevantADL*	31,370	0.3305	0.4704	0	1
*RelevantODL*	31,370	0.2163	0.4117	0	1
*RelevantOTN*	31,370	0.1050	0.3066	0	1
*StrictlyRelevantOTN*	31,370	0.0430	0.2029	0	1
*WeaklyRelevantOTN*	31,370	0.0620	0.2412	0	1
*IrrelevantATN*	31,370	0.1113	0.3144	0	1
*IrrelevantATN1*	31,370	0.1025	0.3033	0	1
*IrrelevantATN2*	31,370	0.0088	0.0934	0	1

In 2005, more than 44% of unique country pairs exempt foreign dividends under their domestic law—thus ignoring bilateral DTTs. On the other end of the spectrum, 10.5% of unique country pairs give no relief for foreign taxes. 5.7% of all pairs allow foreign taxes to be deducted as a business expense. Remaining country pairs credit the host withholding tax from the domestic tax liability, with almost 11% of all pairs crediting also the underlying corporate tax. Once we include bilateral tax treaties, the shares of no relief and deduction drop to approximately 9% for the former and 5% for the latter; the percentage of countries using the direct credit method remains stable around 29%; indirect credits’ share rises above 11%; while the use of exemption method increases the most to more than 45%.

In terms of the cheapest connection en route, we observe that for more than 53% of all country pairs the direct connection is the cheapest one. Further, 35.5% achieve the minimum tax distance on an indirect route with one conduit company and 10.7% on an indirect route with two conduits. Overall, 7213 out of 18,906 unique country pairs have a zero tax distance, where there are no repatriation taxes on distributed income. Corporate income is taxed thus only once, at the level of the subsidiary, and there is no economic double taxation.^14^ Close to half, 48.5%, of the zero tax distance connections occur on the direct connection, about 42% on an indirect route with one intermediate country and the remaining 9.7% on an indirect route with two intermediates.

In 2005, 3487 country pairs had an effective DTT.^15^ Out of these, 1500 country pairs had a *RelevantODL* tax treaty to the extent that it reduced the direct tax distance. Among these DTTs, about half, 733, remain relevant once accounting for the possibility of treaty shopping. The overwhelming majority (723 out of 767) of country pairs with an *IrrelevantATN* dummy is at the disadvantage of a cheaper indirect route that involves only one conduit. Finally, more DTTs, 410 against 323 country pairs, cut the direct tax distance to the minimum one in the network rather than below it.

Moving to the last year in our sample, 2012, and leaving again the effect of tax treaties aside, about 9% of all country pairs have no relief for foreign taxes; 5.8% use deduction as the only relief method; 27.1% apply direct credit; approximately 9.8% offer indirect credit; and 49.4% apply exemption. Taking into account bilateral DTTs, the share of the no relief method drops below 8% and that of the deduction method to 5%. At the same time, the shares of all other methods increase to 27.6% in the case of direct credit; 9.4% in the case of indirect credit; and 50.3% for the exemption method.

Focusing again on the cheapest connections in the network, we see that now only 52.1% of the cheapest connections occur on the direct route. This suggests that treaty shopping has gained in importance over the last decade. The use of indirect routes with one conduit company increases to above 37%, whereas indirect routes with two conduits increases to 10.7%. Overall, 8907 out of 18,906 country pairs have a zero tax distance. Among these, 46.1% country pairs have a direct tax distance of 0%; 43.3% country pairs have a zero tax distance on an indirect route with one intermediate country; while the remaining 10.6% zero tax distances are achieved on an indirect route with two intermediates.

Finally, in 2012, 4264 country pairs had an effective DTT. 1903 of them are of the *RelevantODL* treaties and 2361 of the *IrrelevantADL* treaties. Similarly to 2005, about half, 1013, of *RelevantODL* treaties turn irrelevant once accounting for the possibility of treaty shopping. More country pairs are now at a disadvantage of indirect routes involving two intermediate countries (92 out of 1013); the number of country pairs with a *WeaklyRelevantOTN* treaty increases to 579; and the number of country pairs with a *StrictlyRelevantOTN* treaty decreases to 311.

The presented statistics show the surprisingly high fraction of irrelevant tax treaties, both against domestic law and against tax treaties network. Even excluding DTTs concluded between EU countries in light of the particular position of the Parent-Subsidiary Directive, we find that more than 70% of *IrrelevantADL* tax treaties remain in place. This last observation confirms that *IrrelevantADL* DTTs are not specific to the EU, but a network-wide phenomenon. Moreover, it demonstrates the increasing role of domestic legislation in the avoidance of (economic) double taxation and the scale of domestic law changes.^16^

The high fraction of *IrrelevantATN* tax treaties (more than half the number of *RelevantODL*) suggests that a majority of countries does not take into account the entire tax treaty network upon negotiating new DTTs or renegotiating already existing DTTs. Particularly when a more recent tax treaty between two-third countries creates a new tax-minimising indirect route, one could expect more renegotiations of DTTs already in place. In contrast, the once-concluded tax treaties are characterised by a low level of dynamism and infrequent changes.

### Tax treaties, treaty benefit and treaty shopping

While the identification of conduit countries is not the main purpose of this paper (in analogy with Van ’t Riet and Lejour 2018, and Hong 2018), we are interested in the channels along which DTTs reduce taxes on repatriation. Until recently, the preamble to the OECD Model Tax Convention stated a single objective of double tax treaties: to eliminate double taxation with respect to taxes on income and capital. In this way, DTTs were supposed to promote international economic activity. At the same time, for more than half (17,899 out of 30,918) of all country pairs in our sample with an effective DTT, the tax treaty does not reduce taxes on repatriation relative to the conditions under domestic law. Given their objective, is there still a rationale behind these tax treaties?

Over the years, DTTs have come to pursue additional goals such as providing legal certainty, preventing tax discrimination in the state of investment and exchange of information for tax matters. Indeed, Ligthart et al. (2011) show in a gravity framework that while countries sign DTTs primarily to reduce international double taxation, providing a legal instrument for the exchange of information in tax matters is another important objective. In support of a broader function of DTTs, Davies et al. (2009) and Blonigen et al. (2014) show positive treaty effects ascribed to giving legal certainty and establishing guidelines for dispute settlement between tax authorities of the signatory countries. Moreover, the current preamble to the OECD Model Tax Convention adds the prevention of tax avoidance and evasion as additional objectives of double tax treaties. Finally, the scope of DTTs stretches beyond taxes on corporate profit and includes determination of fiscal residence and rules allocating taxing rights for individuals. In light of this, even treaties defined as irrelevant in this paper, are far from being fully “irrelevant” and the role of DTTs is unlikely to diminish in the near future.

**Fig. 2:**
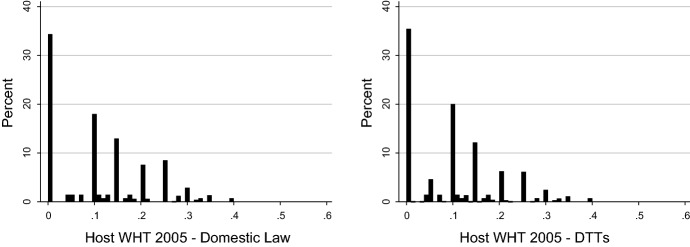
Host-country withholding tax rates 2005

**Fig. 3:**
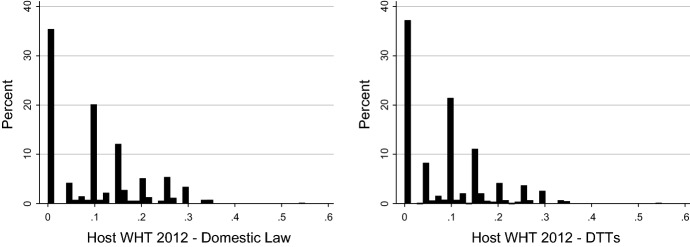
Host-country withholding tax rates 2012

Having addressed the position of irrelevant tax treaties under domestic law, we focus our attention on *RelevantODL* DTTs and the driving forces behind their relevance. Table 3 suggests only a modest improvement in the relief methods once the effect of DTTs is taken into account. The development of the host-country dividend withholding taxes under domestic law and under tax treaties can be seen in Fig. 2 for 2005 and in Fig. 3 for 2012. While about 35% of country pairs have zero withholding at source unilaterally, there is a notable shift towards lower withholding tax rates with DTTs in place, especially towards the 5% and 10% withholding tax rates.

**Fig. 4:**
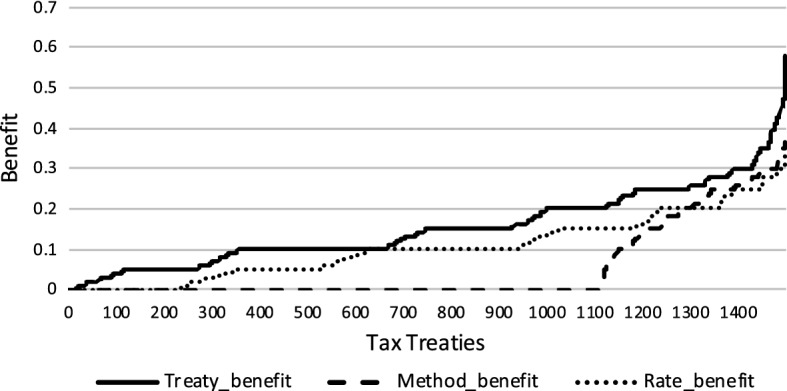
Benefit tax treaties 2005

**Fig. 5:**
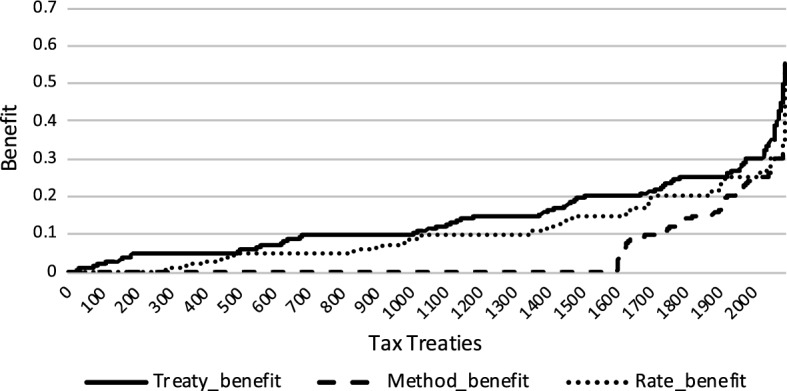
Benefit tax treaties 2012

We also show the treaty benefit (solid line) for all tax treaties effective in the year 2005 in Fig. 4 and all tax treaties effective in the year 2012 in Fig. 5. We then disentangle this treaty benefit into benefit that can be attributed to lower withholding tax rate (dotted line) and benefit that can be attributed to more generous relief methods (dashed line).

Comparing the treaty benefits of lower withholding taxes and more generous relief methods suggests that the reduction in withholding taxes is the driving force behind treaty relevance. However, while in theory the rate and relief method benefits should add up to the total benefit, they actually slightly overestimate it.^17^ We calculate each channel by holding the other factor constant at its domestic value. As a result, if a treaty operates along both margins—by providing a more generous relief method and lower withholding taxes—we are either not able to achieve the full potential of tax treaties or calculate a given benefit twice.

We decide to follow this methodology because it is not known on theoretical grounds whether a certain benefit should be attributed to more generous relief methods or lower withholding taxes. In particular, as long as the home corporate tax rate is higher than the host withholding tax rate, relief by credit method would take away the entire advantage of lower withholding taxes. If a subsequent treaty further reduces the withholding tax rate and at the same time exempts foreign dividends, it cannot be established which effect happens first.

We are also interested in which countries are more likely to have *RelevantODL* and *RelevantOTN* treaties. Logit estimations suggest that relevant tax treaties are positively correlated with the GDP per capita ratio of the home country and negatively correlated with the GDP per capita ratio of the host country.^18^ Not surprisingly, relevant DTTs are negatively correlated with EU membership of home and host countries. However, the correlation is positive with the year of signature of the tax treaty. This might be indicative of countries’ learning effects over the years as well as the tendency over the years to impose higher source withholding taxes and grant less generous unilateral relief methods towards the low-tax jurisdictions.

In terms of the relief methods, we observe different effects depending on whether we look at the domestic method of double tax relief or the one specified in the DTT. Whereas more generous relief methods under domestic law are negatively correlated with relevant tax treaties, the opposite is true for more generous relief methods under DTTs. The underlying mechanism is evident: the more generous the domestic relief method, the lower the domestic tax distance between the host and home states and the less room there is for the treaty to lower the distance even further. A more generous treaty relief method has the potential to reduce home taxation of foreign dividends beyond the unilateral conditions and secures the benefits of lower withholding taxes at source.

We observe an analogous mechanism in the case of source withholding tax rates of dividends. The higher domestic withholding tax rates are, the more likely it is that the treaty will lower these rates. The higher the source withholding tax rate is, the less likely it is that the treaty provides a benefit beyond the conditions of domestic law. In addition, it appears that the marginal effect of domestic and treaty withholding tax rates is stronger than that of domestic and treaty relief methods. This supports the notion presented in Figs. 4 and 5 that the reduction in withholding taxes is the driving force behind treaty relevance.

In the case of *RelevantOTN* treaties, the results are the same except for two variables: the GDP per capita ratio of the home country—which is only weakly significant for *RelevantODL* treaties—and the signature year of the treaty are no longer significant. The significance level and the sign of coefficients of all other variables remain intact. However, the much lower magnitude of domestic and treaty relief methods as well as domestic and treaty dividend withholding taxes suggests that interactions with other treaties in the network are less prone to domestic tax policy choices.

**Fig. 6:**
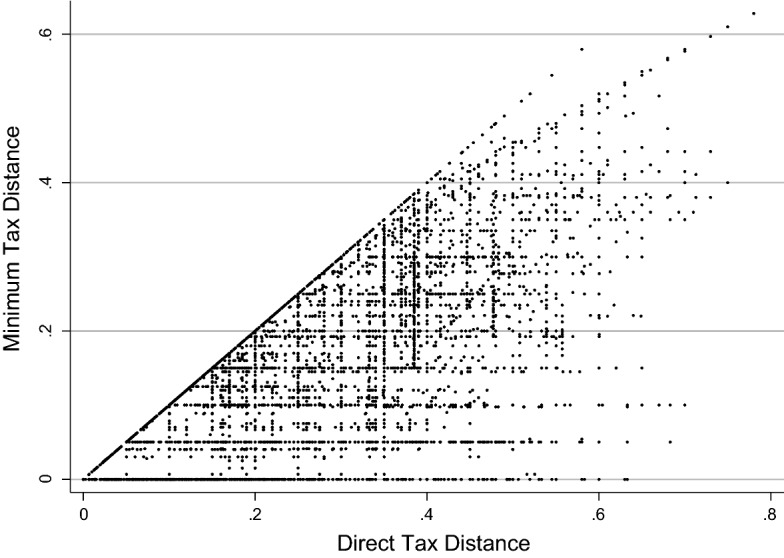
Potential gains from treaty shopping

Finally, Fig. 6 describes the economic consequences of treaty shopping. On the horizontal axis, we plot for every single observation in our panel the direct tax distance in the absence of treaty shopping, *DirectTaxDistance*. On the vertical axis, we show effective taxes paid if instead an indirect route via one or two conduits is chosen. Points along the diagonal exhibit no gains of treaty shopping. All countries where the direct distance is the cheapest route will be along this line. The greater the vertical distance from the diagonal, the bigger the saving due to treaty shopping. We show ample possibilities for treaty shopping, in many cases reducing the actual tax burden to zero.

Figure 6 reveals two interesting patterns. We find a series of vertical lines, which typically reflect individual country pairs, where neither domestic tax regulation nor the DTT have changed, and hence, the tax burden along the direct route remains unchanged. However, subsequent treaties signed with or between third countries have reduced the tax burden along the indirect route, demonstrating how the international DTT network undermines national policy. We also observe that a great deal of our observations occurs along the 5%, 10% and 15% effective tax rates, which reflect the withholding tax rates usually agreed on in DTTs. These are represented by the horizontal lines. Under the exemption system applied by the majority of countries in our sample, the actual tax burden is brought back from the level of domestic withholding tax rates to these common treaty withholding tax rates. Moreover, a significant number of observations are concentrated along the 25%, 30% and 35% direct tax distance, which coincides with the corporate tax rates of many counties. These points comprise all instances where the home country unilaterally offers a foreign tax credit—thereby setting the direct tax distance equal to the domestic corporate tax rate—but the MNEs benefit from tax treaties with a more generous method of double tax relief.

## Estimation methodology and main results

The standard procedure to infer DTT effects on bilateral FDI stocks employs a gravity model and accounts for the presence of a DTT with a dummy variable equal to 1 when a tax treaty is effective between two countries in year *t* and 0 otherwise. We include the variables derived from the network analysis and adopt a Poisson estimator (pseudo-maximum likelihood estimation—PPML). We resort to the PPML estimator as proposed by Santos-Silva and Tenreyro (2006) to account for zero FDI stocks and, more importantly, heteroskedasticity in FDI data (Azémar and Dharmapala 2019). In particular, Santos-Silva and Tenreyro (2006) argue that the standard log-linear OLS approach results in inconsistent coefficient estimates. Mainly because of doubts about the exclusion restriction (Anderson and Yotov 2016), we decide not to follow the formal model of selection proposed by Helpman et al. (2008). Given the large number of fixed effects, we use the *ppml_panel_sg* STATA command (Larch et al. 2017) and estimate the following equation:

11 $$\begin{aligned} \mathrm{FDI}_{ps,t} = \exp [\beta _1T_{sp,t} + \beta _2D_{sp,t} + \beta _3X_{sp,t} + \eta _{s,t} + \theta _{p,t} + \gamma _{sp}] + \epsilon _{s,t} \end{aligned}$$

where $T_{sp,t}$ is a vector of tax rates or tax differentials, composed of *DirectTaxDistance* and its interaction terms; $D_{sp,t}$ is a vector of tax treaty dummies that describe the international tax network; and $X_{sp,t}$ is a vector of control variables, in our case only *BIT*. Finally, $\eta _{s,t}$ and $\theta _{p,t}$ denote the time-varying host country, respectively home-country fixed effects, $\gamma _{sp}$ captures country-pair fixed effects and $\epsilon _{s,t}$ is the Poisson error term. If the dependent variable is in levels, the coefficient can be interpreted analogous to a log-linear estimation. A unit increase in the regressor will lead to a $100(e^\beta -1)$ percentage increase in the dependent variable.

Time-varying host-country and home-country fixed effects absorb any between-country variation over time. Similarly to Baier and Bergstrand (2007) and Anderson and Yotov (2016), we use country-pair fixed effects, as prescribed by Wooldridge (2010), to address DTT endogeneity and control for the physical distance between the host country and the home country. Accordingly, any effect of DTTs identifies only the within-country-pair variation over time.

We present our main results in Table 5. First, we replicate results of the prior literature in column (1), using just a *BIT* and a *Treaty* dummy, as well as the direct tax distance to measure the tax burden on FDI, alongside our host, home and country-pair-fixed effects. The goal of this exercise is to validate that our later results are not driven by sample size, time series, estimation methodology or an omitted variable bias, and as such are directly comparable to the results of previous literature. The lack of significant results for the generic *Treaty* dummy confirms this premise.

**Table 5:** Effects of double tax treaties

	(1)	(2)	(3)	(4)	(5)	(6)	(7)
*BIT*	$-$ 0.0121	$-$ 0.00914	$-$ 0.0127	$-$ 0.0125	$-$ 0.0141	$-$ 0.0144	$-$ 0.0118
	(0.0996)	(0.0940)	(0.0910)	(0.0907)	(0.0898)	(0.0894)	(0.0880)
*DirectTaxDistance*	0.0440	0.157	0.245	0.243	0.313	0.317	0.411
	(0.320)	(0.316)	(0.326)	(0.339)	(0.315)	(0.325)	(0.353)
*Treaty*	0.0514						
	(0.0734)						
*IrrelevantADL*		$-$ 0.0201	$-$ 0.0179	$-$ 0.0180	$-$ 0.0192	$-$ 0.0189	$-$ 0.0441
		(0.0758)	(0.0751)	(0.0752)	(0.0757)	(0.0758)	(0.0871)
*RelevantODL*		0.137*					
		(0.0720)					
*RelevantOTN*			0.166**		0.160**		
			(0.0688)		(0.0685)		
*StrictlyRelevantOTN*				0.164**		0.164**	0.349***
				(0.0809)		(0.0809)	(0.112)
*WeaklyRelevantOTN*				0.167**		0.159**	0.166**
				(0.0706)		(0.0701)	(0.0812)
*IrrelevantATN*			0.109	0.109			
			(0.0854)	(0.0858)			
*IrrelevantATN1*					0.0597	0.0592	0.112
					(0.0827)	(0.0828)	(0.125)
*IrrelevantATN2*					0.210*	0.210*	0.428***
					(0.110)	(0.110)	(0.152)
*DTDxIrrelevantADL*							0.176
							(0.469)
*DTDxStrictlyRelevantOTN*							$-$ 1.638**
							(0.778)
*DTDxWeaklyRelevantOTN*							0.503
							(0.843)
*DTDxIrrelevantATN1*							$-$ 0.769
							(0.928)
*DTDxIrrelevantATN2*							$-$ 3.286**
							(1.389)
Observations	31,370	31,370	31,370	31,370	31,370	31,370	31,370
R-squared	0.992	0.993	0.992	0.992	0.993	0.993	0.993
Home-year FE	Yes	Yes	Yes	Yes	Yes	Yes	Yes
Host-year FE	Yes	Yes	Yes	Yes	Yes	Yes	Yes
Country-pair FE	Yes	Yes	Yes	Yes	Yes	Yes	Yes

Dependent variable: FDI stocks (2005–2012). Robust standard errors clustered by country pairs in parentheses

***, ** and *significance at the 1, 5 and 10% confidence level

The results change dramatically, once we replace the *Treaty* dummy with our measures of relevance in columns (2)–(7). While the variables of the previous specification (1) remain insignificant, we now observe several interesting effects. Whereas the generic *Treaty* dummy did not have a statistically significant effect on bilateral FDI, this effect differs between *IrrelevantADL* and *RelevantODL* DTTs. As shown in column (2), only tax treaties that offer investors a financial advantage over the conditions under domestic law, i.e. reduce the direct tax distance, exhibit a statistically significant impact on bilateral FDI at the 10% significance level. A *RelevantODL* DTT increases FDI by about 14.7%, while *IrrelevantADL* DTTs show no effect.

The results of the dummies derived from the network analysis reveal an even more complex mechanism behind the effects of tax treaties on bilateral FDI. We allow for the possibility of treaty shopping in column (3) and find that among the group of *RelevantODL* DTTs, only tax treaties that are also relevant against the network lead to more FDI (by almost 18%). Tax treaties can only have an impact on foreign investment if they reduce the tax burden with respect to the existing global network of double tax treaties, i.e when they are relevant. Any treaty between third countries can affect the relevance of a national treaty network, which implies that countries lose some of their capabilities to set tax policy due to treaty shopping.

With a *t* test rejecting any difference in the coefficients between *StrictlyRelevantOTN* and *WeaklyRelevantOTN* dummies in column (4), we focus our attention on column (5). The results here suggest that the effects of *IrrelevantATN* DTTs are not universal and differ depending on the number of conduit countries needed to achieve the tax-minimising indirect route. Correspondingly, column (5) demonstrates the importance of accounting for the potential costs to treaty shopping. In particular, *IrrelevantATN2* DTTs may provide sufficient benefits on the direct route to overcome the overly complicated tax-minimising indirect route. However, these benefits may not be enough to overcome the tax advantage of indirect routes with only one conduit country.^19^

Indeed, if a bilateral DTT is at the disadvantage of an indirect route with only one intermediate jurisdiction, *IrrelevantATN1*, this treaty shows no effect on home- to host-country FDI. It may very well be that a structure of one conduit is typical for the reference category (absence of a treaty), and thus, this case cannot be distinguished from a case of an *IrrelevantATN1* treaty. Meanwhile, tax treaties that are at the disadvantage only against an indirect route with two intermediate jurisdictions show strongly significant positive effect on FDI. Structures that are complicated and costly may thus be avoided against a simpler direct route, once a DTT becomes available. While the coefficient of *IrrelevantATN2* is higher than the one of *RelevantOTN*, this difference is statistically insignificant.

We confirm the results of columns (4) and (5) in column (6), by splitting *RelevantOTN* DTTs into *StrictlyRelevantOTN* and *WeaklyRelevantOTN* DTTs, and *IrrelevantATN* DTTs into *IrrelevantATN1* and *IrrelevantATN2* DTTs simultaneously. The statistical significance and magnitude of all variables remain the same.

Finally, we measure the elasticity of FDI with respect to the tax distance by interacting our treaty dummies and the direct tax distance (*DTD*), column (7). The *StrictlyRelevantOTN* and *WeaklyRelevantOTN* treaty dummies remain statistically significant, as does the dummy controlling for a cheaper route that involves two conduits. In particular, for a direct tax distance of 0%, *WeaklyRelevantOTN* DTTs increase FDI on the intensive margin by about 18%, whereas the effect is between 42% and 53% for *StrictlyRelevantOTN* and *IrrelevantATN2* DTTs. In addition, we now observe a statistically significant coefficient for the interaction term of the direct tax distance with *StrictlyRelevantOTN* and the *IrrelevantATN2* dummy. The interaction terms expose a further 8.1% (*StrictlyRelevantOTN*) and 9.6% (*IrrelevantATN2*) increase in FDI for a 10 percentage points decrease in the direct tax distance.

Overall, we believe that our results shed light on the so far empirically mixed results in the prior literature. Specifically, we are confident that our results highlight the importance of recognising the international tax system as a network and show distinct effects of tax treaties by distinguishing their position vis-á-vis the domestic law and all other treaties in the network. Tax treaties can only have an impact on foreign direct investment if they reduce the tax burden below the conditions under domestic law and their final impact will depend on their relevance in the existing global network of double tax treaties. Any treaty between third countries can affect the relevance of a single tax treaty, which implies that countries lose some of their capabilities to set tax policy due to treaty shopping.

## Robustness tests

We base the robustness tests in Tables 6, 7 and 8 focusing on the full model presented in Table 5 column (6) and the model with interaction terms presented in Table 5 column (7). Our main model controls for simultaneous effects of trade policy through a dummy for bilateral investment treaties. In Table 6, columns (1) to (4), we control also for other forms of trade policy, which, if introduced at the same time as DTTs, could bias our tax treaty estimates.^20^ In particular, we consider a dummy indicator taking the value of unity if both home and host countries are a member of WTO and zero otherwise. While Table 6, columns (1) and (2) show a statistically significant and positive effect of WTO membership on FDI stocks, our main coefficients of interest are not affected by the introduction of this variable, in terms of both their significance level and magnitude.

**Table 6:** Robustness tests I

	WTO		RTA		Negative FDI = 0	
	(1)	(2)	(3)	(4)	(5)	(6)
*DirectTaxDistance*	0.315	0.413	0.276	0.372	0.318	0.404
	(0.325)	(0.354)	(0.334)	(0.363)	(0.324)	(0.351)
*IrrelevantADL*	$-$ 0.0154	$-$ 0.0387	$-$ 0.0213	$-$ 0.0477	$-$ 0.0175	$-$ 0.0420
	(0.0761)	(0.0875)	(0.0759)	(0.0874)	(0.0757)	(0.0869)
*StrictlyRelevantOTN*	0.166**	0.353***	0.158*	0.348***	0.167**	0.346***
	(0.0811)	(0.112)	(0.0813)	(0.113)	(0.0806)	(0.112)
*WeaklyRelevantOTN*	0.161**	0.170**	0.157**	0.164**	0.161**	0.163**
	(0.0702)	(0.0813)	(0.0701)	(0.0814)	(0.0697)	(0.0808)
*IrrelevantATN1*	0.0615	0.116	0.0594	0.113	0.0626	0.0893
	(0.0829)	(0.126)	(0.0827)	(0.125)	(0.0825)	(0.127)
*IrrelevantATN2*	0.212*	0.432***	0.209*	0.428***	0.213*	0.424***
	(0.110)	(0.152)	(0.110)	(0.152)	(0.110)	(0.152)
*DTDxIrrelevantADL*		0.167		0.190		0.181
		(0.469)		(0.469)		(0.468)
*DTDxStrictlyRelevantOTN*		$-$ 1.640**		$-$ 1.674**		$-$ 1.587**
		(0.778)		(0.774)		(0.773)
*DTDxWeaklyRelevantOTN*		0.482		0.508		0.561
		(0.843)		(0.842)		(0.844)
*DTDxIrrelevantATN1*		$-$ 0.778		$-$ 0.776		$-$ 0.427
		(0.927)		(0.927)		(0.992)
*DTDxIrrelevantATN2*		$-$ 3.295**		$-$ 3.297**		$-$ 3.194**
		(1.388)		(1.391)		(1.394)
*WTO*	0.210**	0.215**				
	(0.0919)	(0.0910)				
*RTA*			0.0284	0.0318		
			(0.0683)	(0.0667)		
Observations	31,370	31,370	30,177	30,177	32,007	32,007
R-squared	0.993	0.993	0.993	0.993	0.993	0.993
Home-year FE	Yes	Yes	Yes	Yes	Yes	Yes
Host-year FE	Yes	Yes	Yes	Yes	Yes	Yes
Country-pair FE	Yes	Yes	Yes	Yes	Yes	Yes

Dependent variable: FDI stocks (2005–2012). Bilateral investment treaties control variable not presented. Robust standard errors clustered by country pairs in parentheses

***, ** and *significance at the 1, 5 and 10% confidence level

Next, we control for simultaneous introduction of regional trade agreements (*RTAs*) in Table 6, column (3) and (4). We extend our main model with a dummy taking the value of unity if the observed country-pair is part of a regional trade agreement. Being statistically insignificant, the additional control does not affect our tax treaty estimates. Overall, the additional control variables confirm the robustness of our main results with respect to different forms of trade policy.

Because the PPML estimator does not allow for negative values of FDI stocks, we treat these observations as missing in our main model. While negative FDI flows are economically meaningful and represent disinvestments in the host economy, negative FDI stocks are generally the consequence of accounting methods (Gouel et al. 2012). We confirm our results replacing the negative FDI stocks with a zero in Table 6 columns (5) and (6).

Cheng and Wall (2005) point out that “fixed-effects estimations are sometimes criticised when applied to data pooled over consecutive years on the grounds that dependent and independent variables cannot fully adjust in a single year’s time.” (p.8). To address this concern, we follow Anderson and Yotov (2016) and estimate our model using either only the years 2005, 2007, 2009 and 2011 or only the years 2006, 2008, 2010 and 2012, which is comparable to the 3-years interval in Trefler (1993). We present these results in Table 7, columns (1) to (4). While the results differ between the two intervals, especially the *StrictlyRelevantOTN* dummy and its interaction term with *DirectTaxDistance* are consistently robust.

**Table 7:** Robustness tests II

	Intervals 2005–2011		Intervals 2006–2012		Tax havens	
	(1)	(2)	(3)	(4)	(5)	(6)
*DirectTaxDistance*	$-$ 0.0565	0.0463	0.689*	0.782*	$-$ 0.154	0.272
	(0.343)	(0.367)	(0.390)	(0.434)	(0.496)	(0.548)
*IrrelevantADL*	$-$ 0.0313	$-$ 0.0684	$-$ 0.0152	$-$ 0.0327	$-$ 0.0920	$-$ 0.0943
	(0.121)	(0.143)	(0.0801)	(0.0873)	(0.0935)	(0.114)
*StrictlyRelevantOTN*	0.0821	0.295**	0.268**	0.441***	0.108	0.344**
	(0.107)	(0.137)	(0.107)	(0.144)	(0.116)	(0.164)
*WeaklyRelevantOTN*	0.105	0.125	0.239**	0.217**	0.0728	0.144
	(0.0983)	(0.113)	(0.0938)	(0.103)	(0.104)	(0.122)
*IrrelevantATN1*	$-$ 0.0219	$-$ 0.0682	0.164	0.276*	$-$ 0.0265	0.150
	(0.105)	(0.149)	(0.104)	(0.143)	(0.106)	(0.161)
*IrrelevantATN2*	0.125	0.287*	0.282**	0.426**	$-$ 0.0301	0.276*
	(0.112)	(0.154)	(0.140)	(0.206)	(0.112)	(0.143)
*DTDxIrrelevantADL*		0.400		0.00535		0.00759
		(0.517)		(0.540)		(0.571)
*DTDxStrictlyRelevantOTN*		$-$ 1.601*		$-$ 1.838*		$-$ 1.779**
		(0.879)		(0.976)		(0.762)
*DTDxWeaklyRelevantOTN*		$-$ 0.547		1.784*		0.156
		(1.071)		(0.959)		(0.804)
*DTDxIrrelevantATN1*		0.715		$-$ 1.879*		$-$ 2.047**
		(1.009)		(1.064)		(1.039)
*DTDxIrrelevantATN2*		$-$ 2.514*		$-$ 1.973		$-$ 3.748***
		(1.455)		(2.111)		(1.340)
Observations	15,043	15,043	15,024	15,024	21,357	21,357
R-squared	0.993	0.993	0.993	0.993	0.994	0.995
Home-year FE	Yes	Yes	Yes	Yes	Yes	Yes
Host-year FE	Yes	Yes	Yes	Yes	Yes	Yes
Country-pair FE	Yes	Yes	Yes	Yes	Yes	Yes

Dependent variable: FDI stocks (2005–2012). Bilateral investment treaties control variable not presented. Robust standard errors clustered by country pairs in parentheses

***, ** and *significance at the 1, 5 and 10% confidence level

**Table 8:** Robustness tests III

	Treaty Lead		FDI Diversion		Lagged FDI	
	(1)	(2)	(3)	(4)	(5)	(6)
*DirectTaxDistance*	0.115	0.213	0.277	0.359	0.423	0.464
	(0.340)	(0.385)	(0.325)	(0.352)	(0.287)	(0.298)
*IrrelevantADL*	$-$ 0.0478	$-$ 0.0793	$-$ 0.00269	$-$ 0.0329	0.0116	$-$ 0.0194
	(0.110)	(0.127)	(0.0758)	(0.0876)	(0.0805)	(0.0911)
*StrictlyRelevantOTN*	0.0891	0.251**	0.175**	0.366***	0.213**	0.381***
	(0.0982)	(0.122)	(0.0815)	(0.114)	(0.0948)	(0.122)
*WeaklyRelevantOTN*	0.0898	0.0714	0.156**	0.160**	0.200**	0.189**
	(0.0886)	(0.102)	(0.0704)	(0.0815)	(0.0846)	(0.0940)
*IrrelevantATN1*	0.00396	$-$ 0.00736	0.0757	0.133	0.114	0.162
	(0.104)	(0.144)	(0.0814)	(0.122)	(0.0974)	(0.134)
*IrrelevantATN2*	0.135	0.268	0.225**	0.445***	0.183	0.403**
	(0.124)	(0.171)	(0.111)	(0.152)	(0.123)	(0.165)
*DTDxIrrelevantADL*		0.175		0.239		0.275
		(0.489)		(0.471)		(0.419)
*DTDxStrictlyRelevantOTN*		$-$ 1.487*		$-$ 1.667**		$-$ 1.484*
		(0.777)		(0.772)		(0.787)
*DTDxWeaklyRelevantOTN*		0.958		0.633		1.258
		(0.921)		(0.830)		(0.809)
*DTDxIrrelevantATN1*		$-$ 0.0920		$-$ 0.845		$-$ 0.790
		(0.968)		(0.903)		(0.890)
*DTDxIrrelevantATN2*		$-$ 1.905		$-$ 3.282**		$-$ 3.216**
		(1.536)		(1.396)		(1.371)
*LeadTreaty*	0.0654	0.0658				
	(0.0555)	(0.0556)				
*FDIConduit*			7.25e$-$07***	7.38e$-$07***		
			(2.43e$-$07)	(2.43e$-$07)		
*LagFDI*					1.21e$-$06***	1.18e$-$06***
					(3.06e$-$07)	(3.07e$-$07)
Observations	26,714	26,714	31,370	31,370	26,057	26,057
R-squared	0.993	0.993	0.993	0.993	0.994	0.994
Home-year FE	Yes	Yes	Yes	Yes	Yes	Yes
Host-year FE	Yes	Yes	Yes	Yes	Yes	Yes
Country-pair FE	Yes	Yes	Yes	Yes	Yes	Yes

Dependent variable: FDI stocks (2005–2012). Bilateral investment treaties control variable not presented. Robust standard errors clustered by country pairs in parentheses

***, ** and *significance at the 1, 5 and 10% confidence level

As explained by Braun and Weichenrieder (2015), firms may invest in tax havens not only because of low tax rates, but also for non-tax reasons, such as secrecy. Schjeldrup (2016) provides complementary reasons for the demand for secrecy by multinational enterprises. However, as pointed out by van’t Riet and Lejour (2018) tax havens are not crucial in treaty shopping structures. Moreover, our set of time-varying host- and home-country fixed effects should capture any unobservable reasons to invest in tax haven jurisdictions. To confirm that our results are not biased by the presence of tax havens, we conduct a separate analysis excluding all of them. We present the results in Table 7, columns (5) and (6). Except for the model without interaction terms, all of our variables remain significant. Overall, we conclude that our findings are robust to the presence of tax havens.

An inherent concern in the empirical literature estimating the effects of tax treaties is the issue of potential reverse causality between DTTs and the level of FDI (Egger et al. 2006; Baker 2014). In line with previous literature, we address this concern by adding the *future* level of DTTs to the regression model, *LeadTreaty* (Baier and Bergstrand 2007; Wooldridge 2010; Yotov et al. 2016). In the panel context here, if DTT changes are strictly exogenous to FDI stock changes, *LeadTreaty* should be uncorrelated with the concurrent FDI stock.

We add a *generic* lead treaty dummy out of identification concerns. In particular, the lead treaty dummy should control for reverse causality between *new* DTTs and the concurrent level of FDI stocks and not between the specific type of DTT as identified in the network analysis and the FDI position. If DTTs change their type due to changes in domestic legislation, separate lead dummies for each type of DTT would wrongly interpret this as a new tax treaty. For example, if a *RelevantODL* DTT turns irrelevant following a reduction of the withholding tax rate under the domestic law, a *LeadIrrelevantATN* dummy would pick this up as a new tax treaty, which is not the case. Therefore, a generic lead treaty dummy allows us to effectively control only for the newly concluded tax treaties.

As shown in Table 8, columns (1) and (2), the coefficient estimates of *LeadTreaty* are economically small and not significantly different from zero. Hence, the results confirm no “feedback effects” from changes in FDI stocks to DTT changes.

In the presence of FDI diversion via a third country, the bilateral FDI stocks are not independent of each other. For a given capital stock, the availability of a shorter (cheaper) indirect route leads to lower FDI stock on the direct route and vice versa. Ideally, we want to observe what fraction of bilateral FDI stocks is diverted via a conduit country to an ultimate host destination. However, the available FDI data do not allow for that degree of identification. This leads to the concern that the estimated increase in FDI might result from ending a diversion of FDI due to past treaty shopping, without affecting the total inward stock of FDI in the host country.

To accommodate this concern, we follow a similar approach to Azémar and Dharmapala (2019). Specifically, we extend our main model with a control variable corresponding to the total inward FDI stock in the observed host country from OECD countries having a tax treaty with the observed home country—*FDIConduit*.^21^ The inclusion of this variable allows us to see whether FDI received from the observed home country substitutes for FDI received from any potential conduit country. Table 8, columns (3) and (4) show a positive and statistically significant coefficient on *FDIConduit*. If anything, these results tend to indicate a complementary relationship between total host inward FDI in potential conduit countries and bilateral FDI, although the effect is small in magnitude. Most importantly, however, the estimated coefficients of the main variables of interest remain unchanged, both in terms of their statistical significance and economic magnitude.^22^

In all of our baseline estimations, we follow the standard practice in the empirical literature on the effects of DTTs and use FDI stocks as the dependent variable (Blonigen and Davies 2004; Egger et al. 2006; Azémar and Dharmapala 2019). In case there were a lot of inertia in foreign direct investment, changes in the treaty network might only affect new FDI, suggesting the use of FDI flows instead. We do this by including one-year lagged FDI as a dependent variable in Table 8, columns (5) and (6), and the results remain robust.^23^ Moreover, the use of FDI stocks has the additional value added that one does not lose meaningful observations associated with negative FDI flows.

Finally, we add two further variables to our analysis. Similarly to Hong (2018), we create a dummy variable that indicates whether an indirect route exhibits a shorter tax distance than the direct route, labelled *NetworkConnection*. The use of a conduit obviously identifies treaty shopping and this is irrespective of a potential DTT between the home country and the host country. If the indirect route is the cheapest one, we measure the reduction in the tax burden due to treaty shopping, *NetworkBenefit*. Noteworthy, *NetworkBenefit* measures the tax benefit in an international setting. In particular, MNEs face a complex investment decision and the choice of the investment channel depends also on other non-tax factors. In this regard, *NetworkBenefit* captures the opportunity cost of not using the tax-preferred indirect path relative to a direct investment. If firms react to higher relative tax cost of investing directly from home to host countries, we expect the bilateral FDI to decrease with the size of *NetworkBenefit*.

We extend each of our models (1) to (7) with the two network variables.^24^ Surprisingly, neither of them turns out significant. However, since *NetworkConnection* measures the within-country-pair variation over time due to a change in the shortest investment channel from a direct route to an indirect one, it captures just the opposite effect of our *StrictlyRelevantOTN* and *WeaklyRelevantOTN* dummies, but independent of a country-pair having a DTT or not. With this variable turning insignificant once we account for the heterogeneous impact of tax treaties, we can actually confirm that the FDI increases due to *StrictlyRelevantOTN* and *WeaklyRelevantOTN* DTTs. Firms do not disinvest when an indirect route becomes cheaper, but increase investment following a *StrictlyRelevantOTN* or *WeaklyRelevantOTN* DTT. The fact that *NetworkBenefit* also turns out insignificant may be due to fixed effects absorbing the between-country variation over time.

## Conclusions

This paper has investigated the effects of taxes on foreign direct investment. Despite the growing number of contributions in the literature, the empirical evidence on the effects of double tax treaties on bilateral FDI has so far been inconclusive. This paper provides evidence that this may be due to the fact that surprisingly many tax treaties are irrelevant. In order to avoid high host country withholding taxes on outgoing passive income, many multinational companies divert FDI via a third country with a more favourable tax treaty. Nevertheless, the vast majority of the existing literature treats DTTs as a binary variable, thereby ignoring their complexity and their domestic and international interactions.

Our study addresses this gap in the literature and analyses the effects of double tax treaties allowing for treaty shopping and for a differential effect of DTTs. We differentiate DTTs with respect to their relevance in terms of reduction of the overall tax burden to or below the one under domestic law and to and below the minimum one in the network. We define as irrelevant a DTT that will not provide investors with a financial benefit and distinguish whether the indirect route involves one or more conduits.

Our main result is that only relevant DTTs will lead to an increase in direct bilateral FDI, and we can estimate an upper bound of the effect at roughly 18%. Treaties that are irrelevant with respect to domestic law and treaties that are irrelevant with respect to an alternative indirect route that involves only one conduit do not alter direct bilateral FDI. We can attribute the latter result to the power of treaty shopping. Whereas the direct effective tax rate between two countries has no direct effect on direct bilateral FDI, we find that an increase in the tax burden will reduce FDI for strictly relevant DTTs.

We also observe two sources of effects on bilateral FDI in cases where the alternative involves two conduits and the indirect route is thus complicated and more costly. In this case firms apparently prefer a slightly more expensive direct route to redirecting investment via an indirect route. However, a strong reaction to an increase in the tax burden indicates that firms will tolerate only a modest premium.

We demonstrate that tax treaties can only impact foreign investment if they reduce the tax burden with respect to the existing global network of double tax treaties, i.e. when they are relevant. Any treaty between third countries can affect the relevance of a national treaty network, which implies that countries lose some of their capabilities to set tax policy.

## Footnotes

1 This includes countries that operate a territorial tax system where all foreign profits are exempt and countries that adopt worldwide taxation with a participation exemption for foreign dividends. Some countries exempt only 95% of the received dividends with, typically, a foreign tax credit or no relief for the remaining 5% of the dividends. Other countries exempt 100% of the received dividends, but disallow the deduction of certain costs connected with the participation. To simplify the analysis, we ignore these distinct characteristics.

2 We are limited to this time span, as domestic tax data are not available systematically for the years before 2005, whereas data on FDI are not yet available for the years after 2012.

3 This includes the applicability of the EU Parent-Subsidiary Directive.

4 If under the tax treaty between country A and country B dividends are subject to a withholding tax rate that is equal to the withholding rate under domestic law, any change in the domestic rate affects directly the rate under the tax treaty.

5 FDI data present one additional challenge: whereas our measure of tax distance consists of the cost of channelling corporate income from one country to another in terms of taxes to be paid, firms may use debt financing instead of dividend. To the extent that FDI stocks include debt financing, the relationship between FDI stocks and tax distance becomes weaker and we are less likely to find significant results. Moreover, as DTTs do not create tax liabilities, they cannot increase the tax cost of debt financing. Therefore, the identification of *relevant* tax treaties is not biased by including tax treaties that leave investors worse off.

6 The Floyd–Warshall algorithm calculates total taxes over the indirect route taking into account only the nominal taxes in the intermediate jurisdictions. Assume that the home country relieves double taxation through the indirect credit method, whereas the intermediate jurisdiction exempts foreign dividends. Under the Floyd–Warshall algorithm, the home country credits the underlying corporate tax at the full nominal tax rate. However, the actual tax rate on the dividends in the intermediate jurisdiction is 0%. van’t Riet and Lejour (2018) tackle this problem by substituting the nominal corporate tax rate in any intermediate jurisdiction with a worldwide average corporate tax rate.

7 VBA is an implementation of Microsoft’s programming language Visual Basic 6 and it is built into most Microsoft Office applications, including Microsoft Excel.

8 Assuming a subsidiary engaged in an active course of business allows us to ignore potential anti-abuse provisions targeted against treaty shopping. While this presents a limitation, the significant differences in the subjective and objective scope of these provisions make it fairly impossible to treat them in a systematic way. Moreover, whereas the OECD has put an effort in combating treaty shopping, its actions and recommendations are only recent and later than our sample years (see for instance OECD, 2015). Finally, the effectiveness of anti-treaty shopping rules proves disputable, as shown in the joined cases *Deister Holding* and *Juhler Holding* where the Court of Justice of the European Union declared the German provisions as not compatible with the EU freedom of establishment. To the extent that we overestimate the potential for treaty shopping, our results should be interpreted as the upper bound.

9 Our sample includes the following tax havens listed in Dyreng and Lindsey (2009): Aruba, Bahamas, Bahrain, Barbados, Bermuda, Botswana, British Virgin Islands, Brunei, Cayman Islands, Cyprus, Gibraltar, Guernsey, Ireland, Isle of Man, Jersey, Latvia, Lebanon, Liechtenstein, Luxembourg, Macau, Maldives, Malta, Mauritius, Monaco, Panama, Seychelles, Singapore, Switzerland, Uruguay, US Virgin Islands.

10 Note that this differs from subject to *a* tax condition. Here, we assume that all companies are subject to tax.

11 This implies looking at 136 different routes with a single conduit for each of our 138 home countries to each of our host countries, and 136 times 135 different routes for two conduits, effectively comparing 350 million cases.

12 One of the possible indirect routes is through the UK and Bolivia: the tax distance between Belgium and the UK is 0% (no withholding tax in Belgium and participation exemption in the UK) and so is the tax distance between the UK and Bolivia (no withholding tax in the UK, while foreign dividends are not subject to tax in Bolivia). Finally, the tax distance between Bolivia and Argentina is 12.5% resulting in an overall cost of 12.5%.

13 Note that the tax distance between two countries can be asymmetric, i.e. it is more expensive to distribute dividends from country *A* to country *B* than vice versa.

14 Note that the same outcome is achieved under the EU Parent-Subsidiary Directive.

15 The effective date of tax treaties can differ between the two signatory countries. This explains the uneven number of effective DTTs.

16 Unfortunately, due to limited historical data on domestic withholding tax rates and methods of double tax relief, we are unable to confirm this empirically.

17 It is equally likely that the rate and relief methods underestimate the total benefit.

18 Results available upon request.

19 We would like to thank an anonymous referee for this remark.

20 All of our additional control variables come from Head et al. (2010).

21
Azémar and Dharmapala (2019) study the effects of tax sparing provisions included in DTTs on FDI. They account for FDI diversion by controlling for the total FDI from the observed home country to OECD countries having a tax sparing provision with the observed host country.

22 In alternative specifications, which are available upon request, we confirm the main results by not restricting the potential conduit countries to OECD members only; and by controlling for the total outward FDI stock from the observed home country to OECD countries having a tax treaty with the observed host country, using the exact same approach as Azémar and Dharmapala (2019).

23 While significantly limiting the sample, the results hold also when including two-year or three-year lagged FDI and the estimates remain available upon request.

24 Results available upon request.
